# Genotoxic Effects of Culture Media on Human Pluripotent Stem Cells

**DOI:** 10.1038/srep42222

**Published:** 2017-02-08

**Authors:** Megha Prakash Bangalore, Syama Adhikarla, Odity Mukherjee, Mitradas M. Panicker

**Affiliations:** 1 National Centre for Biological Sciences (TIFR), Bangalore - 560065, India; 2 Manipal University, Madhav Nagar, Manipal 576104, Karnataka, India; 3 Institute for Stem Cell Biology and Regenerative Medicine, Bangalore 560065, India

## Abstract

Culture conditions play an important role in regulating the genomic integrity of Human Pluripotent Stem Cells (HPSCs). We report that HPSCs cultured in Essential 8 (E8) and mTeSR, two widely used media for feeder-free culturing of HPSCs, had many fold higher levels of ROS and higher mitochondrial potential than cells cultured in Knockout Serum Replacement containing media (KSR). HPSCs also exhibited increased levels of 8-hydroxyguanosine, phospho-histone-H2a.X and p53, as well as increased sensitivity to γ-irradiation in these two media. HPSCs in E8 and mTeSR had increased incidence of changes in their DNA sequence, indicating genotoxic stress, in addition to changes in nucleolar morphology and number. Addition of antioxidants to E8 and mTeSR provided only partial rescue. Our results suggest that it is essential to determine cellular ROS levels in addition to currently used criteria i.e. pluripotency markers, differentiation into all three germ layers and normal karyotype through multiple passages, in designing culture media.

The capability of human pluripotent stem cells (HPSCs) to self-renew as well as differentiate into all cell types makes them valuable for therapy, in understanding early developmental processes and to model many human diseases. These unique properties of stem cells are regulated by a number of complex and specialized processes which require that their genomic integrity be stable and maintained. Various studies have indicated that the levels of reactive oxygen species in mouse and human pluripotent stem cells (PSCs) are significantly lower than their differentiated counterparts^1^,^2^,^3^. This has been hypothesized as a way to protect cellular components i.e. lipids, protein, RNA and DNA from oxidative damage. They are also reported to have increased abilities to repair their DNA to maintain genomic stability^4^,^5^,^6^,^7^,^8^,^9^.

Over the years, several studies have aimed at making ‘clinically useful’ HPSCs. The source of somatic cells and the process of reprogramming have been examined to determine sources of genomic variation^10^,^11^,^12^,^13^. Extensive research has also gone into optimizing the ideal culture conditions to maintain and propagate HPSCs leading to the development of different substrates and media which are ‘chemically defined and xeno-free’, can support feeder-free cultures of HPSCs, show lower batch-batch variation and increased ease of handling^14^,^15^,^16^,^17^,^18^,^19^,^20^,^21^,^22^. In these studies, the ‘quality’ of stem cells has been defined by robust expression of pluripotency markers, capability to differentiate into all the three germ layers, established by teratoma formation or *in vitro* differentiation, and the presence of normal karyotypes after multiple passages. Efficient derivation of ESC and iPSC lines in these media has also been another criterion. Curiously, mitochondrial activity and ROS levels of established PSCs during routine culture in different media *per se* have not been addressed. Perhaps, this has been, in part, due to early studies that have indicated that HPSCs depend on glycolysis and not on oxidative phosphorylation, and that PSCs, in general, exhibit low ROS levels^2^,^3^,^23^,^24^. A variety of media formulations now available, have antioxidants such as glutathione (GSH), Vitamin C and N-acetyl cysteine (NAC) which have been empirically determined to improve cultures though the cellular ROS levels or mitochondrial potential in these cultures have not been examined. In an earlier study, we had identified lipid droplets containing retinyl esters as a marker unique to the ‘primed’ pluripotent state. We had also observed that these droplets were present in cells cultured in Knockout Serum Replacement (KSR) containing media but not in Essential 8 (E8) and mTeSR media^25^. This suggested that the metabolic state i.e. lipid metabolism, of HPSCs in these two media were different and led us to examine other aspects of HPSCs in these media, in more detail. We observed significant changes in the nuclear and nucleolar morphology of cells in the three media. Changes in the morphology of nucleoli which are known to be markedly affected by stress^26^,^27^,^28^ led us to investigate the metabolic activity of HPSCs in different media which often impacts ROS levels and mitochondrial potential. Our study shows that HPSCs in E8 and mTeSR media have higher levels of ROS and mitochondrial potential when compared to KSR media. Associated with these, were higher levels of markers for double stranded DNA breaks (DSBs) and increased sensitivity to γ-irradiation induced DSBs. The RNA in HPSCs cultured in these two media also exhibited increased levels of 8-hydroxy guanosine in the nucleoli. The increased oxidative stress seen in E8 and mTeSR media would certainly affect their long term culture and genomic status. Associated with the higher ROS levels were also increased number of single nucleotide variations (SNVs) in the genomic DNA. While karyotypic changes, which would report large changes in genomic DNA have been used as a surrogate for genomic integrity, SNVs caused by these media have not been reported.

Media commonly used to culture HPSCs have been assumed to be equivalent with respect to genotoxicity and differ primarily in their ease of use, the number of components and their xeno-free status. Although media can have very pronounced metabolic effects^29^, ROS levels and mitochondrial potential of HPSCs cultured in different media have not been examined which we show here, are important.

## Results

### Nuclei and nucleoli of HPSCs cultured in E8 and mTeSR media are morphologically distinct from those in KSR medium

**Figure 1:**
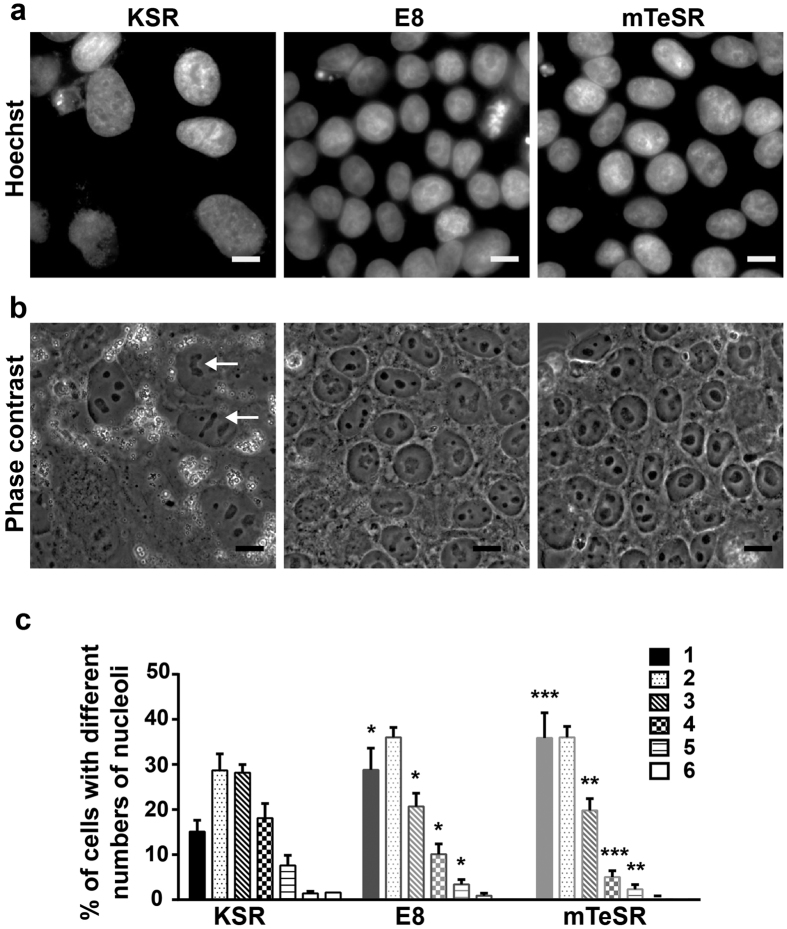
Nuclear and nucleolar morphologies of HPSCs cultured in KSR, E8 and mTeSR media are different. (**a**) Hoechst 33342 staining of ADFiPS showing larger nuclei in KSR compared to E8 and mTeSR media. (**b**) Bright field images of ADFiPS corresponding to their respective Hoechst images in (**a**) showing reticulate & multiple nucleoli per nucleus in KSR, fewer reticulate nucleoli and higher numbers of single nucleoli per nucleus in E8; large and round, mostly single nucleolus per nucleus in mTeSR. (**c**) Quantification of the percentage of nuclei with different number of nucleoli for HPSCs in the three media. 1–6 refers to the number of nucleoli per nucleus. All the scale bars represent 10 μm. Pooled data from all the four cell lines represented as mean ± SEM. Unpaired t-test with Welch’s correction. ****p < 0.0001, ***p < 0.001, **p < 0.01, *p < 0.05. n ≥ 10 for all quantitative data and each ‘n’ represents a sample of at least 50 nuclei.

HPSCs cultured in E8 and mTeSR media exhibited very different nuclear and nucleolar morphologies from those seen in KSR (1:1 mixture of conditioned and unconditioned media). This was also observed in images from the literature^30^ and from those provided by the manufacturers (http://www.stemcell.com/en/Products/Popular-Product-Lines/mTeSR-Family.aspx). The differences in the size and shape of cells in the three media were significant and also reproducible (Fig. 1a & b, Supplementary Figure S1 and Table 1). A systematic analysis using nuclear (Hoechst 3342) staining showed that the nuclei in KSR appeared larger than nuclei in E8 and mTeSR. This was evident with the nuclear cross-sectional area of HPSCs being highest in KSR (Fig. 1a and Table 1) even though the total nuclear volume in all three media remained constant (Table 1). Further analysis determined that the height of nuclei was the least in KSR and significantly higher in E8 and mTeSR (Table 1). Also, the nuclei in KSR had more elliptical/lobular nuclear cross-sections while in E8 and mTeSR, were more circular as determined by the eccentricity values of the nuclear cross-sections (Table 1). Overall, cells in E8 and mTeSR were smaller and more compactly arranged than in KSR, which was also evident from the number of nuclei per unit area of culture surface (Table 1). These observations are in agreement with a recent study which has also reported that HPSCs in E8 are more compact when compared to KSR^29^. These differences were consistent irrespective of cell densities in the three media suggesting that the cell density does not impact nuclear size and shape (Supplementary Figure S1).

HPSCs in these three media also exhibited differences in nucleolar morphology and numbers. In mTeSR, HPSCs predominantly had one or two large circular nucleolus or nucleoli per nucleus while in KSR, multiple reticulate nucleoli per nucleus were seen (Fig. 1b & c and Supplementary Figures S2 & S4). Cells in E8 also had increased numbers of nuclei with 1–2 predominantly circular nucleoli (Fig. 1b & c and Supplementary Figures S2 & S4). In other words, more than 65% of HPSC nuclei in E8 and mTeSR had one or two nucleoli and less than 35% with multiple nucleoli (≥3) whereas in KSR, 35% of nuclei had one or two nucleoli and about 65% with multiple nucleoli (Fig. 1c). Reticulate nucleoli were seen most frequently in KSR followed by E8 and almost completely absent in mTeSR (Fig. 1b and Supplementary Figures S2 & S4). HPSCs cultured for 48 hours in conditioned E8 and mTeSR also had nucleolar morphologies similar to those in unconditioned E8 and mTeSR (Supplementary Figure S2).

E8 and mTeSR have been in use for over five and ten years respectively, but surprisingly, these characteristics have not been reported/examined earlier.

### HPSCs cultured in E8 and mTeSR have higher ROS levels and mitochondrial potential

**Figure 2:**
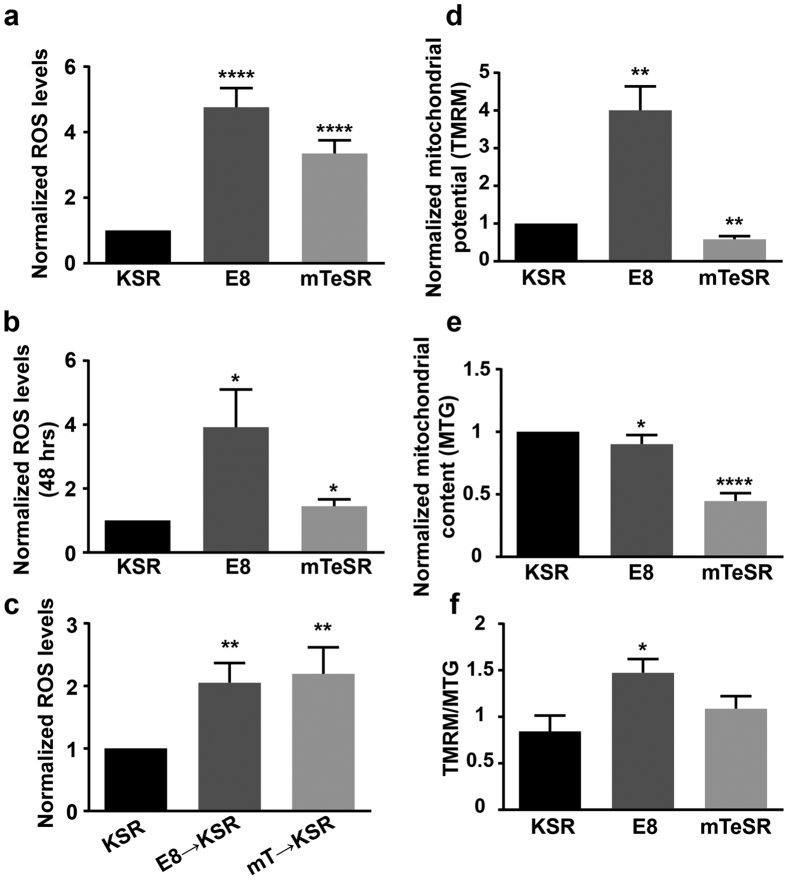
ROS levels and mitochondrial potential of HPSCs in E8 and mTeSR media are higher than in KSR. Quantification of DCFDA fluorescence values (ROS levels) of HPSCs cultured in the three media for (**a**) ≥3 passages (**b**) 48 hours and (**c**) in KSR media for 5–8 days after three passages in E8 and mTeSR; n ≥ 10, n = 3 and n = 6, respectively. Quantification of mitochondrial potential expressed as (**d**) TMRM fluorescence/cell and (**f)** TMRM/MTG fluorescence; n ≥ 5 and n = 4 respectively. (**e**) Quantification of mitochondrial content - MTG fluorescence; n = 7. Normalized data represent values normalized with respect to KSR. Pooled data from all the four cell lines represented as mean ± SEM. Unpaired t-test with Welch’s correction. ****p < 0.0001, ***p < 0.001, **p < 0.01, *p < 0.05.

Nuclear morphology has been related to gene expression^31^ and the nucleolus is known to be affected by stress. Since there were pronounced media-dependant changes in nucleolar morphology and numbers, we determined if the cells were stressed to different extents in the three media. We found that ROS levels, which induces cellular stress at high levels, indeed varied with media. ROS levels of HPSCs, estimated by measurement of DCFDA fluorescence of single cells in suspension, were approximately 5 and 3 fold higher in E8 and mTeSR respectively than in KSR (Fig. 2a). High ROS values were seen within 48 hours of shifting cultures from KSR to E8 and mTeSR (Fig. 2b). Interestingly, ROS levels of HPSCs cultured in E8 or mTeSR for three or more passages, did not decrease to the levels typically seen in KSR even after five to eight days when shifted to KSR (Fig. 2c). HPSCs cultured in conditioned E8 and mTeSR media for 48 hours also showed high ROS levels similar to unconditioned E8 and mTeSR media (Supplementary Figure S2). Our data suggests that HPSCs in E8 and mTeSR are subject to very high ROS levels as compared to KSR and that the ROS levels are not easily reversed. This also suggests that substantial metabolic changes occur in these two media which do not revert immediately.

A striking feature in both E8 and mTeSR is the high concentration of bFGF (100 ng/ml) which is also reported to increase ROS^32^. Hence, we examined the effect of high bFGF concentrations on ROS levels in KSR. When KSR was supplemented with high concentrations of bFGF, increase in ROS levels were observed in 48 hours, although they were not comparable to levels seen in E8 or mTeSR suggesting that either high bFGF concentrations in E8 and mTeSR are not the primary cause of increase in ROS levels in E8 and mTeSR or that components of KSR mitigate its effect on ROS levels (Supplementary Table S1).

The high ROS levels led us to examine mitochondrial metabolism of HPSCs in these media. HPSCs are primarily dependent on glycolysis in spite of the presence of well-developed mitochondria. ATP generation in HPSCs is not dependent on oxidative phosphorylation due to high levels of UCP2, an uncoupling protein^33^ and consequently, mitochondrial potential values are believed to be lower. Measurement of mitochondrial potential of HPSCs in the three media showed that mitochondrial potential expressed as TMRM fluorescence per cell was ~3.7 fold higher in E8 and ~0.5 fold lower in mTeSR when compared to that in KSR (Fig. 2d). We also examined the mitochondrial content (MTG fluorescence) of HPSCs in the three media (Fig. 2e). HPSCs in mTeSR had ~half the mitochondrial content when compared to KSR and E8, which in turn showed marginal differences.

Mitochondrial potential when normalized to mitochondrial content and expressed as TMRM fluorescence per unit mitochondrial mass, was higher in mTeSR as compared to KSR although not statistically significant (Fig. 2f). However, mitochondrial potential computed both as TMRM fluorescence per cell and TMRM fluorescence per unit mitochondrial mass, were higher in E8 compared to KSR (Fig. 2d and f).

### Higher ROS levels in HPSCs in E8 and mTeSR results in increased nucleic acid damage

**Figure 3:**
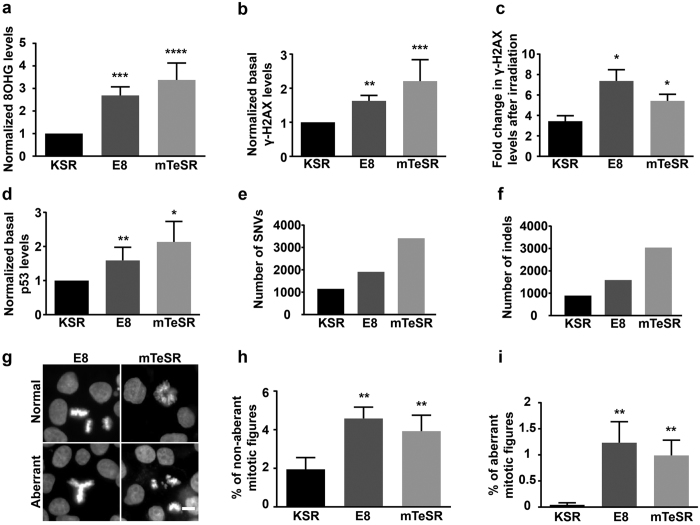
HPSCs in E8 and mTeSR media show higher nuclei acid damage when compared to KSR. Quantification of (**a**) normalized nuclear 8-hydroxyguanosine levels; n = 9, (**b**) normalized basal γ-H2AX levels – DSBs; n ≥ 5, (**c**) fold increase in DSBs after γ-irradiation; n ≥ 3, (**d**) normalized basal p53 levels; n ≥ 4, of HPSCs in the three media. (**e**) Total number of SNVs (**f**) insertions and deletions seen in HuES9 cells cultured in the three media for three passages. (**g**) Representative images of normal and aberrant mitotic figures seen in E8 and mTeSR. Quantification of percentage of (**h**) total mitotic figures and (**i**) aberrant mitotic figures seen in HPSCs in the three media; n = 12. Normalized data represent values normalized with respect to KSR. All the scale bars represent 10 μm. Pooled data from all the four cell lines represented as mean ± SEM. Unpaired t-test with Welch’s correction. ****p < 0.0001, ***p < 0.001, **p < 0.01, *p < 0.05.

ROS generated as a by-product of cellular metabolism can be a major source of DNA damage. Since HPSCs in E8 and mTeSR had substantially higher ROS levels, we measured the extent of oxidative damage in these cells by probing for nuclear 8-hydroxyguanosine (8OHG). HPSCs in mTeSR had the highest levels of nuclear 8OHG followed by E8 while cells in KSR had negligible levels (Fig. 3a and Supplementary Figure S3). Thus, higher levels of ROS in E8 and mTeSR were indeed associated with increased oxidative damage. Double stranded breaks (DSBs), which are known to occur at a very low frequency in routine cultures, were also substantially higher in cells cultured in E8 and mTeSR (Fig. 3b) as measured by γ-H2AX immunofluorescence. Additionally, sensitivity to γ-irradiation, was also higher in HPSCs cultured in E8 and mTeSR when compared to KSR (Fig. 3c). Furthermore, p53 which is known to accumulate in the nucleus in response to DNA damage in HPSCs^34^,^35^ was observed to be higher in the nuclei of HPSCs cultured in E8 and mTeSR when compared to that in KSR (Fig. 3d). Since cell density and the method of passaging influence genomic stability in HPSCs^36^,^37^, cells at different cell densities (50–90%) were analysed. The method of passaging was also kept constant (0.5 mM EDTA) for all the three media to ensure that these factors did not contribute to our results.

The levels of DNA damage were also determined by exome sequencing of one of the cell lines (HuES9) separately cultured in the three media and compared to the human genome sequence. The data showed that there were increased numbers of SNVs in HPSCs cultured in E8 (1.7 times) and mTeSR (3 times) when compared to KSR (Fig. 3e and Table 2). These included both existing and novel SNVs (Table 2). The total number of insertions and deletions (indels) were also higher in E8 (1.4 times) and mTeSR (3.4 times) than in KSR (Fig. 3f and Table 2). The distribution of these SNVs and indels in the various regions of the exome were not dramatically different between HPSCs in the three media for most of the changes. The percentage of indels that resulted in frameshift variations and ‘in-frame’ deletions, however were higher in both E8 and mTeSR while the indels resulting in ‘in-frame’ insertions were higher in mTeSR but not in E8 when compared to KSR (Table 2).

Higher numbers of mitotic figures were also seen in HPSCs in E8 and mTeSR when compared to KSR and were evident in both fixed and live cells (Fig. 3g & h and Supplementary Figures S1 & S4). This may reflect the higher proliferation rate of HPSCs in E8 and mTeSR reported in the literature^18^ or it may be due to more cells undergoing mitotic arrest in E8 and mTeSR due to DNA damage. In addition to the increased number of mitotic figures, the number of aberrant mitotic figures were also higher in E8 and mTeSR (Fig. 3g and i). Representative aberrant mitotic figures observed in E8 and mTeSR are shown in Supplementary Figure S3. The normal mitotic figures observed in KSR are also shown in Supplementary Figure S3.

These observations suggest that HPSCs in E8 and mTeSR are not only subjected to increased basal DNA damage but are also more sensitive to genotoxic treatments e.g. γ-irradiation.

### Addition of antioxidants to E8 and mTeSR provides only partial rescue from genotoxic stress

**Figure 4:**
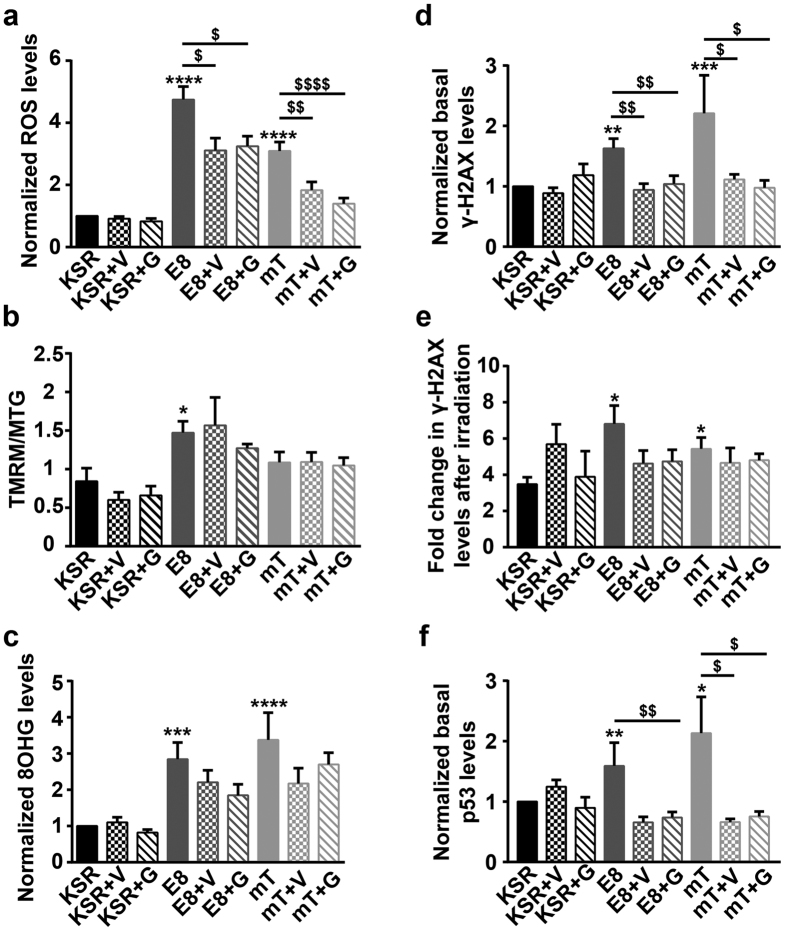
Vitamin-C and glutathione (GSH) partially reduce the effects of E8 and mTeSR media on HPSCs. Quantification of (**a**) normalized ROS levels; n ≥ 10, (**b**) normalized mitochondrial potential; n = 4, (**c**) normalized nuclear 8OHG levels; n = 6, (**d**) normalized basal DSBs (γ-H2AX); n ≥ 5 (**e**) fold increase in DSBs after γ-irradiation; n = 4 and (**f**) normalized p53 levels; n ≥ 4 for HPSCs cultured in the three media. Normalized data represent values normalized with respect to KSR. V: Vitamin-C, G: Glutathione. Pooled data from all the four cell lines represented as mean ± SEM. Unpaired t-test with Welch’s correction. ****p < 0.0001, ***p < 0.001, **p < 0.01, *p < 0.05. ^$^Represent p-values with respect to their untreated controls.

Higher levels of ROS in E8 and mTeSR associated with increased DNA damage led us to investigate the effect of increasing the levels of antioxidants in the media. Glutathione which is absent in E8, and Vitamin C - two commonly used antioxidants in HPSC culture were used^13^,^38^. ROS levels decreased significantly in E8 and mTeSR with the addition of antioxidants but not to the levels observed in KSR (Fig. 4a). Mitochondrial potential however, was not affected with the addition of antioxidants (Fig. 4b). We also examined the effects of a few other known antioxidants which are reported to decrease ROS levels in PSCs (Supplementary Table S1). None of these compounds, decreased the ROS levels of HPSCs grown in E8 and mTeSR, even after three passages.

8OHG levels also decreased with the addition of antioxidants but the values were neither significantly different from their respective untreated controls nor reached the levels seen in KSR (Fig. 4c). Basal γ-H2AX levels however, showed significant decrease with antioxidants and reached values similar to that in KSR (Fig. 4d). Sensitivity to γ-irradiation showed a decreasing trend with the addition of antioxidants to E8 and KSR but the values were not statistically significant from their respective untreated controls (Fig. 4e). Finally, basal p53 values, showed a significant decrease with antioxidants (Fig. 4f). These results suggest that higher levels of ROS in E8 and mTeSR were responsible for increased DNA damage in HPSCs and that addition of antioxidants at the concentrations mentioned here, was only partially effective.

### Nucleolar morphology can act as an indicator of genotoxic stress

**Figure 5:**
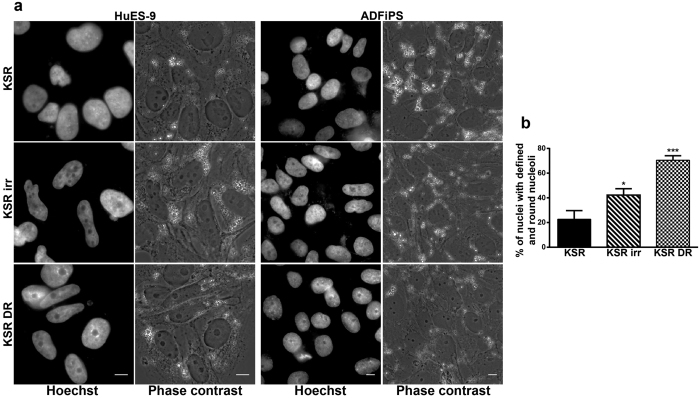
Nucleolar morphology can act as “stress reporter” in HPSCs. (**a**) Representative Hoechst-stained and corresponding phase contrast images of two HPSC lines cultured in KSR media and treated with genotoxic stress – i.e., irradiation (irr) and doxorubicin (DR) showing clear, distinct and rounded nucleoli in KSR treated with γ-irradiation and doxorubicin while reticulate and less defined nucleoli in untreated KSR. (**b**) Quantification of percentage of clear and distinct nucleoli in untreated KSR and KSR with genotoxic treatments; n = 6. All scale bars represent 10 μm. Pooled data from all the four cell lines and represented as mean ± SEM. Unpaired t-test with Welch’s correction. ***p < 0.001, *p < 0.05.

HPSCs in E8 and mTeSR, which have higher ROS levels and associated nuclei acid damage than cells in KSR, possess distinct nucleolar morphologies i.e. rounded and non-reticulate nucleoli unlike in KSR. Since nucleoli are known to be ‘stress sensors’, we examined the change in nucleolar morphology of HPSCs cultured in KSR after subjecting them to genotoxic stress. On treatment of HPSCs in KSR with doxorubicin for three hours, 70.4% of nuclei showed distinct and rounded nucleoli as opposed to only 22.6% in untreated HPSCs (Fig. 5a and b). HPSCs exposed to γ-irradiation also showed a higher proportion of nuclei with distinct and rounded nucleoli (42.2%) within an hour (Fig. 5c). Thus, nucleolar morphology in HPSCs responds to genotoxic stress and within short time frames. However, the number of nucleoli per cell did not decrease as dramatically, with these treatments, unlike the shape.

At the concentrations of the antioxidants used, we also did not observe a reversal of the nucleolar morphology of HPSCs in E8 and mTeSR to that in KSR, reinforcing the observation that even with increased levels of antioxidants in E8 and mTeSR, the rescue from oxidative stress was only partial. This may be due to ROS values of HPSCs cultured in E8 and mTeSR remaining high even after being transferred back to KSR media for five to eight days, suggesting a strong metabolic shift. These results also suggest that the nucleolar morphology can serve as an easily observable indicator for HPSCs undergoing stress.

## Discussion

The culture of HPSCs for regenerative medicine and as model systems to study development and disease, rests on the genomic integrity of the cells through repeated passages. Our initial observations that HPSCs cultured in E8 and mTeSR exhibit differential lipid metabolism and are strikingly different, morphologically, from those cultured in KSR, led us to compare HPSCs grown in these three media. The choice of media that we examined in this study was, in part, dictated by their widespread use. HPSCs grown in E8 and mTeSR have become increasingly popular because of their limited, defined and xeno-free components. These media have been tested through multiple passages using a few criteria i.e. stable expression of pluripotency markers, ability to differentiate into all three germ layers (either *in vitro* or through teratoma formation), stable karyotype and also permissiveness in deriving embryonic and induced pluripotent stem cells. Pluripotent stem cells (PSCs) are reported to have low levels of reactive oxygen species (ROS), and the reduced environment seen in these cells has been attributed as a protective mechanism to prevent nucleic acid, protein and lipid damage but the impact of the various media on ROS levels within these cells have not been evaluated.

Our results indicate that the ROS levels of HPSCs in E8 and mTeSR are many fold higher than in KSR. High levels of ROS are understandably detrimental to the integrity of cells, except in cases where small increases in ROS are known to be a signal for replication and differentiation^39^. The effects of high levels of ROS and associated DNA damage were evident in cells cultured in E8 and mTeSR with increased number of changes in their DNA sequence, presence of a small but significantly higher percentage of cells with both aberrant and non-aberrant mitotic figures and cells being more prone to genomic damage when subjected to radiation. The increased numbers of mitotic figures in HPSCs cultured in E8 and mTeSR may represent ‘arrested’ nuclei in response to high levels of ROS and subsequent DNA damage and remains to be investigated further. Increased DNA damage would result in higher cell death; however, cell viability of HPSCs in the three media did not differ (Supplementary Figure S3). A recent study which examined anueploid HPSCs showed that due to segregation errors, higher percentage of cells exhibited ‘trailing’ DNA during mitosis as opposed to diploid HPSCs^40^. Interestingly, such abnormalities were evident in our HPSCs cultured in E8 and mTeSR but not in KSR (Fig. 3g,i and Supplementary Figure S3). All these observations strongly suggest that cells in E8 and mTeSR media are under severe genotoxic stress and perhaps have defective replication dynamics. Mitochondrial potential is also higher in HPSCs when grown in these two media. HPSCs are known to have low mitochondrial potential due to the high activity of the uncoupling protein, UCP2, which is known to dissipate the mitochondrial potential^33^. It is possible that HPSCs in E8 and mTeSR have lower expression/activity of UCP2 resulting in high mitochondrial potential and remains to be investigated. It is also possible that the higher level of ‘glutaminolysis’ seen in E8^29^ might provide NADH which can subsequently contribute to an increased mitochondrial potential.

Since PSCs give rise to differentiated somatic cells as well as form germ cells, they are expected to be protected from genotoxicity. However, they should also undergo cell death, when the damage is substantial. PSCs have been reported to have more robust oxidative stress defence mechanisms than their differentiated counterparts. So, it is likely that they can survive genotoxic stress up to certain levels beyond which their genomic integrity would be too compromised. It is also likely that in E8 and mTeSR, HPSCs are subjected to high ROS levels but not high enough that they cease to replicate. Though resistant to ROS levels, HPSCs show increased sensitivity to DNA damage, perhaps due to the DNA repair being over-extended even at a basal level. This is reflected by the increase in single nucleotide variations and aberrant mitotic figures seen in HPSCs in E8 and mTeSR. An increase in the number of genomic changes - SNVs and aberrant mitotic figures, are of concern when propagating cells in these media, even for short durations or for derivation of new lines^13^. Moreover, HPSCs grown in E8 or mTeSR, show increased levels of DNA damage markers, namely, γ-H2AX, nuclear p53 and 8-hydroxyguanosine compared to KSR. Cells cultured in E8 and mTeSR for multiple passages are reported to have normal karyotypes^17^,^18^ but that does not exclude the presence of smaller changes in the genome. While these media possess the advantage of having defined components and are feeder-free and xeno-free, our results indicate that they can compromise genomic integrity during cell expansion.

A striking but unreported feature that we noticed in HPSCs cultured in these three media were their differences in nuclear and nucleolar morphology. In E8 and mTeSR, the nuclei of HPSCs are more compact and appear smaller in cross-section while HPSCs in KSR mainly have lobular nuclei and also occupy a significantly larger area. It is to be noted that the compactness of cells in E8 and mTeSR was independent of, the surface area available to expand, as indicated by the arrows in Supplementary Figure S1. HPSCs in E8 and mTeSR were always round and compact within the colonies unlike in KSR. Another striking feature was the difference in the number and shape of the nucleoli of HPSCs cells in these three media as described earlier (Fig. 1b, Table 1 and Supplementary Figures S2 & S4). Nucleoli are known to be stress sensors, get altered due to stress and regulate translation. Reticulate and dispersed nucleoli of HPSCs grown in KSR, upon γ-irradiation or treatment with doxorubicin i.e. subjected to genotoxic stress, become circular and more distinct. This would be in accordance with HPSCs in E8 and mTeSR being under genotoxic stress. The observation that the number and shape of the nucleoli can report on cellular stress levels is useful and can be used to monitor the health of HPSCs.

Interestingly, the ROS levels remained higher even after HPSCs from E8 and mTeSR were transferred back to KSR for five to eight days. It is likely that, with longer periods in KSR, the ROS levels may decrease to the levels seen in cells in continuous culture in KSR. However our results do indicate that the effects of media on the process of ROS generation and metabolism, in general, can be long-lasting. It is known that MEFs secrete various factors into the medium and it is possible that MEF conditioned media may contribute to lower ROS levels in KSR. Hence, we conditioned E8 and mTeSR media on both mitotically inactive (CM BI) and inactive (CM BII) MEFS and assessed ROS levels and nucleolar morphologies of HPSCs cultured in these for 48 hours. HPSCs in conditioned E8 and mTeSR, showed nucleolar morphologies and ROS levels similar to that in unconditioned E8 and mTeSR, respectively (Supplementary Figure S2). So, the lower ROS levels in KSR is unlikely to arise from factors secreted by MEFs.

Antioxidants have been used as supplements in various media for culturing HPSCs. While E8 contains Vitamin C, mTeSR contains both glutathione and Vitamin C. In spite of this, HPSCs in these two media had high ROS levels and increasing the concentration of these antioxidants had only a partial effect in decreasing ROS levels. Addition of various other antioxidants to E8 and mTeSR also only, partially decreased ROS levels (Supplementary Table S1). However, it is interesting to note that, addition of Vitamin C and GSH, reduced the levels of double stranded breaks and nuclear p53 significantly though the ROS levels were not reduced to those seen in KSR (Fig. 4). This suggests that it may be advantageous to increase the level of antioxidants in E8 and mTeSR, further.

It is clear from the lack of lipid droplets in HPSCs cultured in E8 and mTeSR that the lipid metabolism of HPSCs is different in the three media. HPSCs cultured in KSR and E8 have also been shown to have dramatically different lipid metabolism^29^. There have been quite a few studies which have compared different media for culturing HPSCs^20^,^41^,^42^,^43^,^44^,^45^,^46^,^47^ but these studies have focused on the levels of pluripotency markers, karyotypic changes and the differentiation capabilities of HPSCs in the different media to evaluate their relative merits. A very recent study on the effect of cell numbers on genomic stability^37^ has established that minor changes in culture conditions can affect the health and quality of HPSCs, strongly. Our study explores the levels of reactive oxygen species, in particular, and shows that some commonly used and very popular culture media have dramatic and deleterious effects on the health of HPSCs, which is important to keep in mind. It is not apparent at the moment, why the ROS levels are high in these two media compared to KSR. To address this, will require more careful examination and is beyond the scope of this study but our present data strongly suggests that ROS levels should also be investigated in designing new media.

HPSCs destined to be used for therapeutic treatments will require expansion and have to be free of genomic aberrations. Thus, it becomes crucial that the cells be tested for not only gross genomic abnormalities but in addition, for DNA damages such as SNVs and DSBs, which are not routinely tested. These parameters are to be considered in the design of media for culturing HPSCs, particularly for clinical use.

### Experimental procedure

#### Cell culture

- KSR: 20% Knockout Serum Replacement (KSR) in Knockout DMEM supplemented with 5 ng/ml bFGF (Peprotech), Glutamax, Non-Essential Amino Acids, 2-mercaptoethanol, Penstrep (All from Invitrogen)
- E8: Prepared according to manufacturer’s instructions (A1517001)
- mTeSR: Prepared according to manufacturer’s instructions (05850).

Human embryonic stem cells (HuES-7; HuES-9) and human induced pluripotent stem cells (adult dermal fibroblast iPS; neonatal foreskin fibroblast iPS) were cultured without feeders on Matrigel (Corning, #354277) for 1–3 passages before starting the experiments. 1:1 mixture of conditioned and unconditioned KSR media was used as ‘KSR’ on Matrigel. For conditioning the media, KSR, E8 and mTeSR were incubated on MEFs and collected after an incubation period of 24 hours, followed by filtering. Cells were cultured on Vitronectin (Life Technologies, A14700) for E8 and Matrigel, for mTeSR. 0.5 mM EDTA (Life Technologies, 15575020) was used to dissociate cells for regular passaging. For antioxidant experiments, Vitamin C (25 μg/ml) and glutathione (10 μg/ml) were added to the respective media every day during media change. Cells were cultured for at least three passages in the respective media before the qualitative and quantitative assays were done.

#### Measurement of ROS levels

2′,7′-Dichlorofluorescin diacetate (Sigma, D6883) was used at 10 μM. Verapamil hydrochloride (Sigma, V4629) was used at 5 μM with DCFDA to prevent efflux of DCFDA as shown previously^3^. Briefly, cells were incubated with the dye for 20 minutes at 37 °C and 5% CO_2_ after which, they were harvested as cell suspension in 1X PBS for analyses using FACS VERSE. Propidium iodide (PI) (1 μg/ml) was used for selecting live cell population.

Cell viability was calculated as the percent of PI negative cells of the total number of cells acquired.

#### Measurement of mitochondrial potential

Tetramethylrhodamine Methyl Ester Perchlorate -TMRM (Sigma, T668) was used at 10 nM along with Mitotracker green (MTG) (Molecular Probes M7514) at 150 nM. Briefly, cells were incubated with TMRM+MTG for 15 minutes at 37 °C and 5% CO_2_ following which, they were harvested as cell suspension in 1X PBS and analysed using BD FACS Aria. At least 5000 cells were collected for each condition and a minimum of three biological replicates were analysed.

#### Immunostaining and microscopy

Cells were fixed with 4% paraformaldehyde for 15 minutes at room temperature followed by permeabilization with 0.01% Triton-X for 10 minutes. Blocking was done with 10% bovine serum albumin solution for 1 hour at room temperature followed by incubation with primary and secondary antibodies for 1 hour each at room temperature. Hoechst-33342 (1 μ/ml) was used to counter-stain the DNA. Antibodies used were Anti-8 Hydroxyguanosine (Abcam, ab62623; 1:300), Phospho-Histone H2a.X (Ser139) (Cell Signaling Technology, #2577; 1:300), p53 (sc-126; Santa Cruz Biotechnology, INC, 1:100). Alexa Fluor dyes were used as secondary antibodies. Images were acquired on Olympus FV 1000 confocal microscope and analysed using ImageJ and Cell profiler. Geo-mean values of the respective area histograms were used for all the FACS data analyses.

#### Quantification of 8-hydroxyguanosine, phospho-Histone H2a.X and p53 immunostaining

For each of the above experiments, at least five images were acquired per condition per biological sample as image stacks. Fluorescence intensity was calculated per nuclei (equivalent to fluorescence intensity/cell as in FACS analyses) by multiplying the fluorescence intensity per nuclear area by the average depth of the nuclei.

For quantification of fold change in DSBs after irradiation, cells were irradiated with 5 Gy and placed in the incubator for 1 hour after which cells were fixed along with untreated cells. Values were expressed as fluorescence intensity per nuclei of irradiated cells over untreated cells.

#### Quantification of percentage of normal and aberrant mitotic figures

All the cells were fixed and stained with Hoechst for analyses. Normal mitotic figures were identified as nuclei with chromosomes in different mitotic phases as described in an earlier study^6^. Aberrant mitotic figures were identified as nuclei showing trailing DNA segments as described in a recent study^40^ as well as those which showed severely mis-arranged chromosomes (as represented in Supplementary Figure S3). Both were expressed as a percentage of total number of nuclei counted (>1500 for each media).

#### γ-irradiation and doxorubicin treatment

Cells were irradiated with 5 Gy after which they were placed at 37 °C and 5% CO_2_ for an hour. Doxorubicin was added at 1 μM concentration to cells and incubated at 37 °C and 5% CO_2_ for three hours. After both these treatments, cells with the respective untreated controls were fixed with 4% paraformaldehyde and imaged directly or processed further for immunocytochemistry experiments.

#### Exome sequencing and analyses

Genomic DNA was extracted from cells cultured in the three media after three passages by salting out method^48^. DNA libraries were prepared as per manufacturer’s protocol (Nextra rapid capture kit). Pair end sequencing was performed on Illumina Hiseq 2000 platform. The resulting fastq files thus generated were assessed for its quality using fastqc^49^. Low quality reads with the phred score <20 were removed using Prinseq^50^. These were then aligned with human reference genome, hg19 built37 using bwa ver 0.5.9-r16^51^. PCR duplicates were removed using picard tools (http://broadinstitute.github.io/picard/). Indel realignment was performed using GATK (https://www.broadinstitute.org/gatk/). We used the default setting of qulimap ver 2.3.6 52 to generate the QC summary statistics of alignment as given in Supplementary Table S2. SNP, ins/del variants were called by Varscan ver 2.1.1 53 with a coverage of at least 8 and p value of 0.001. These variants were annotated using variant effect predictor^54^.

## Additional Information

**How to cite this article**: Prakash Bangalore, M. *et al*. Genotoxic Effects of Culture Media on Human Pluripotent Stem Cells. *Sci. Rep.* **7**, 42222; doi: 10.1038/srep42222 (2017).

**Publisher's note:** Springer Nature remains neutral with regard to jurisdictional claims in published maps and institutional affiliations.

## Supplementary Material

Supplementary Information

## Figures and Tables

**Table 1:** Nuclear and nucleolar morphologies of HPSCs cultured in KSR, E8 and mTeSR media are different.

	KSR	E8	mTeSR
Normalized nuclear cross sectional area	1	0.62 ± 0.03****	0.57 ± 0.03****
Nuclear height (μm)	6 ± 0.32	9.3 ± 0.25****	11.6 ± 0.43****
Normalized nuclear volume	1	1.05 ± 0.06 ns	1.07 ± 0.07 ns
Eccentricity of nuclear cross sectional area	0.7 ± 0.005	0.61 ± 0.007****	0.6 ± 0.01****
Number of nuclei/ imaging frame	7 ± 0.7	13 ± 1.7***	15 ± 2.6***

Quantification of various parameters defining the nuclei of HPSCs in the three media. All normalized data represent values normalized to those in KSR. Pooled data from all the four cell lines represented as mean ± SEM. Unpaired t-test with Welch’s correction. ^****^p < 0.0001, ^***^p < 0.001, ns: non-significant. n ≥ 10 for all quantitative data and each ‘n’ represents a sample of at least 50 nuclei.

**Table 2:** There is increased DNA damage in HPSCs cultured in E8 and mTeSR when compared to KSR.

HuES9 in different media	KSR	E8	mTeSR
Total nucleotides sequenced	8803716	14683535	26106531
Total SNVs	11480	19097	34128
Novel SNVs	515	799	1373
Known SNVs	10965	18298	32755
% in intronic regions	20	19	21
% in non-coding regions	14	14	13
% of synonymous	7	7	6
% of missense	6	6	5
% of NMD transcript	5	5	5
Total indels	894	1589	3042
Novel indels	28	54	114
Known indels	866	1535	2928
% in intronic regions	25	26	27
% in non-coding regions	12	12	12
% of frameshift variations	1.9	3.3	2.9
% of in-frame insertions	1.5	0.8	3.1
% of in-frame deletions	0.8	1.3	1.2

Summary of exome-sequencing of HuES9 in the three media when compared to the human reference genome, hg19.
