# Supplementary material for: Genotoxic Effects of Culture Media on Human Pluripotent Stem Cells
Source: Sci Rep. 2017 Feb 8;7:42222. doi: 10.1038/srep42222 (PMC5297241; doi:10.1038/srep42222)
Supplement: Supplementary Information [file srep42222-s1.docx]

**Genotoxic Effects of Culture Media on Human Pluripotent Stem Cells**

Megha Prakash Bangalore^1,2,*^, Syama Adhikarla^3^, Odity Mukherjee^3^, Mitradas M. Panicker^1*^

**Supplementary figure S1 related to figure 1: Nuclear size of HPSCs in E8 and mTeSR is smaller than in KSR, irrespective of cell density**


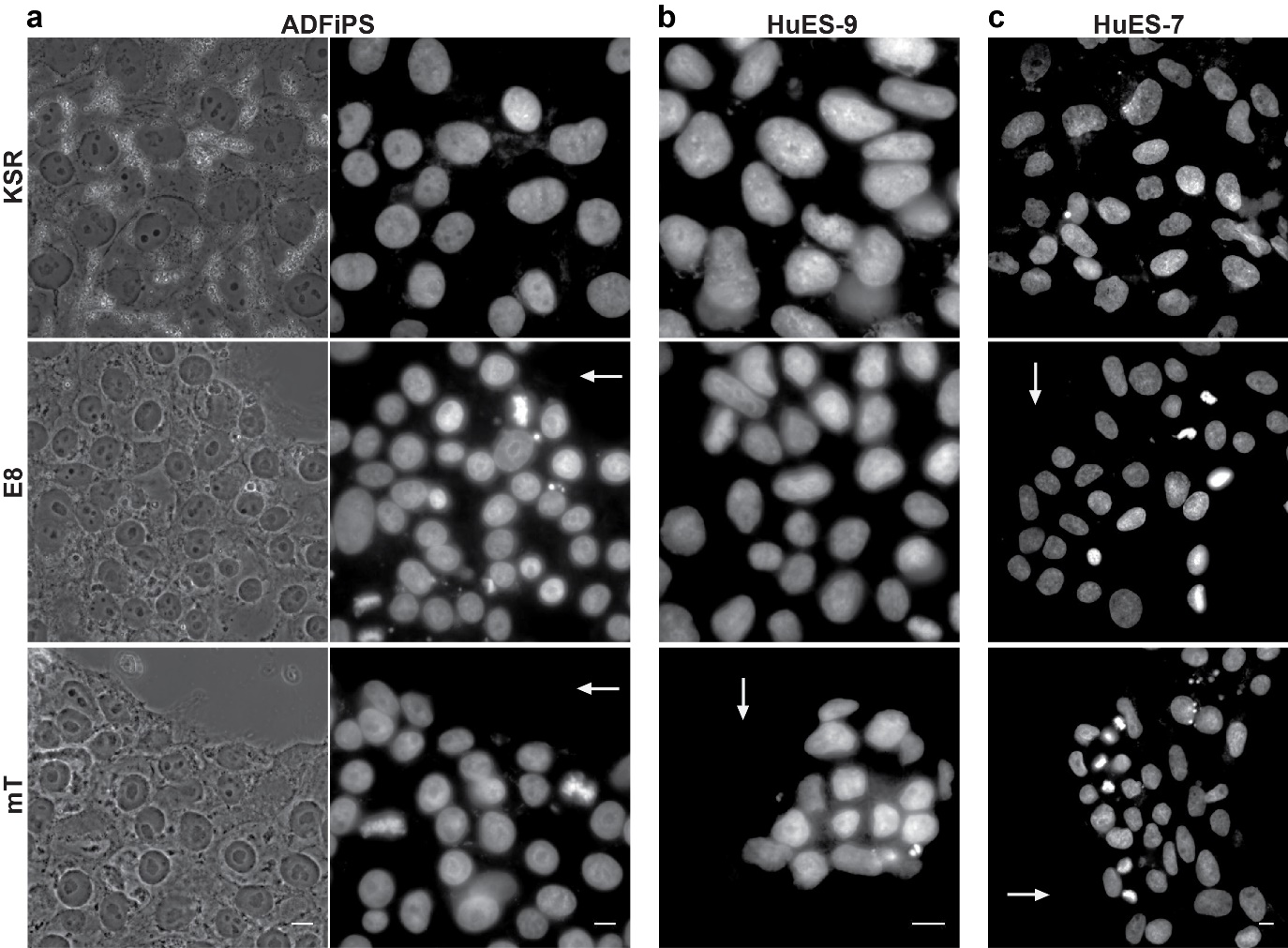


HPSCs in E8 and mTeSR show smaller nuclear size than in KSR. (a) Phase contrast and corresponding Hoechst-stained images of ADFiPS. (b) Hoechst-stained images of HuES-9 (c) Hoechst-stained images of HuES-7 at lower magnification. All panels show the nuclear cross section of HPSCs in KSR being larger than those in E8 and mTeSR. Arrows indicate free space adjacent to the colonies. All scale bars represent 10μm.

**
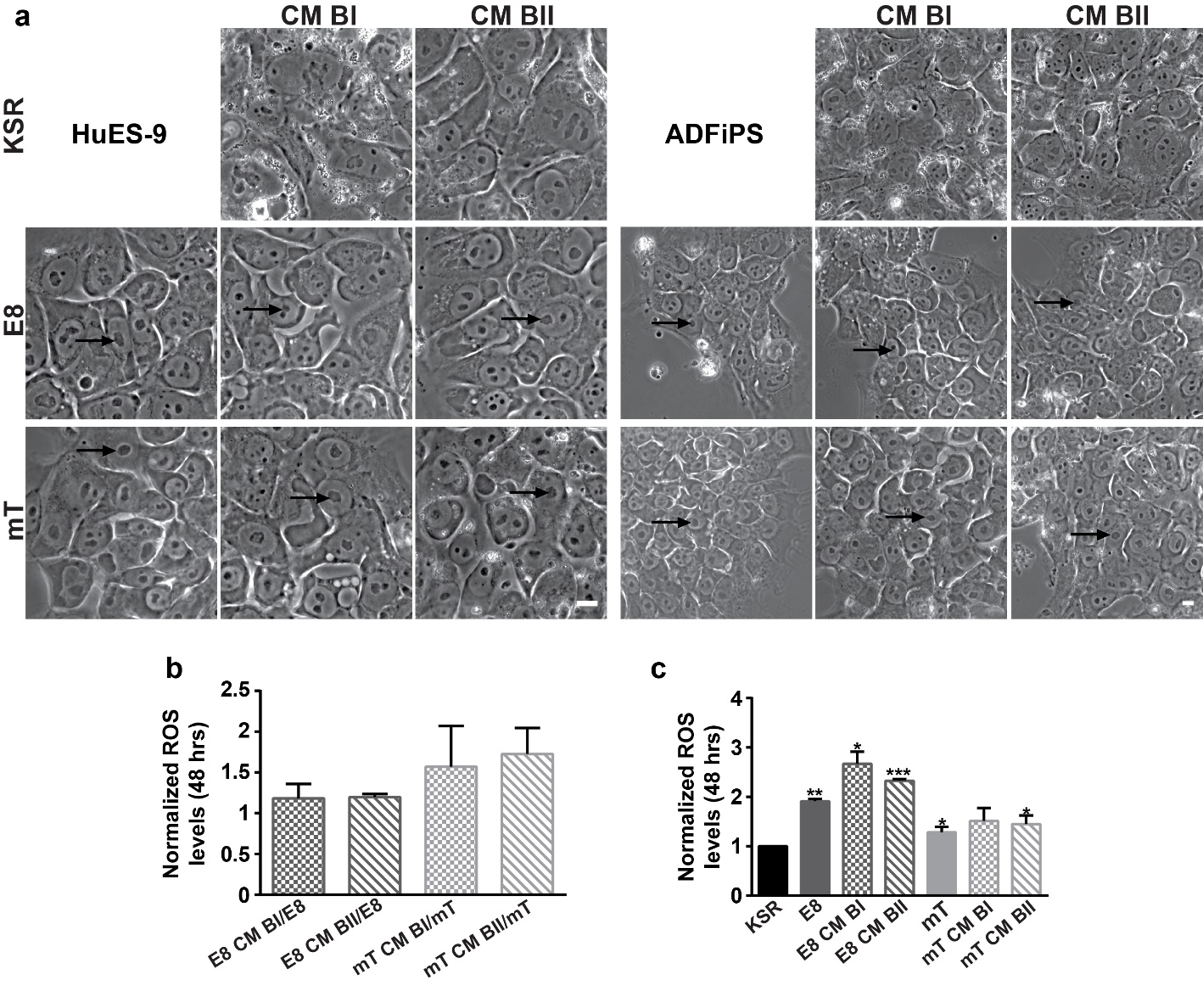
Supplementary figure S2 related to figures 1 & 2: Nuclear & nucleolar morphologies and ROS levels of HPSCs in conditioned E8 and mTeSR are similar to that in unconditioned E8 and mTeSR.**

(a) Nucleolar morphologies of two HPSC lines in conditioned E8 and mTeSR are similar to that in unconditioned E8 and mTeSR but different from those in KSR. Arrows indicate typical single or double rounded, nucleoli per nucleus in both conditioned and unconditioned E8 and mTeSR; CM BI and BII refer to conditioned media batch I and batch II. (b) ROS levels of HPSCs cultured for 48 hours in conditioned E8 and mTeSR expressed after normalizing the values to unconditioned E8 and mTeSR, respectively. (c) ROS levels of HPSCs cultured in unconditioned and conditioned E8 and mTeSR expressed after normalizing to KSR. Pooled data from all the cell lines represented as mean ± SEM. Unpaired t-test with Welch’s correction. *** p<0.001, ** p<0.01, * p<0.05. n=3 for all quantitative data. All scale bars represent 10µm. **Supplementary figure S3 related to figure 3: HPSCs in E8 and mTeSR media show higher nuclei acid damage when compared to that in KSR media but no difference in cell viability**

**
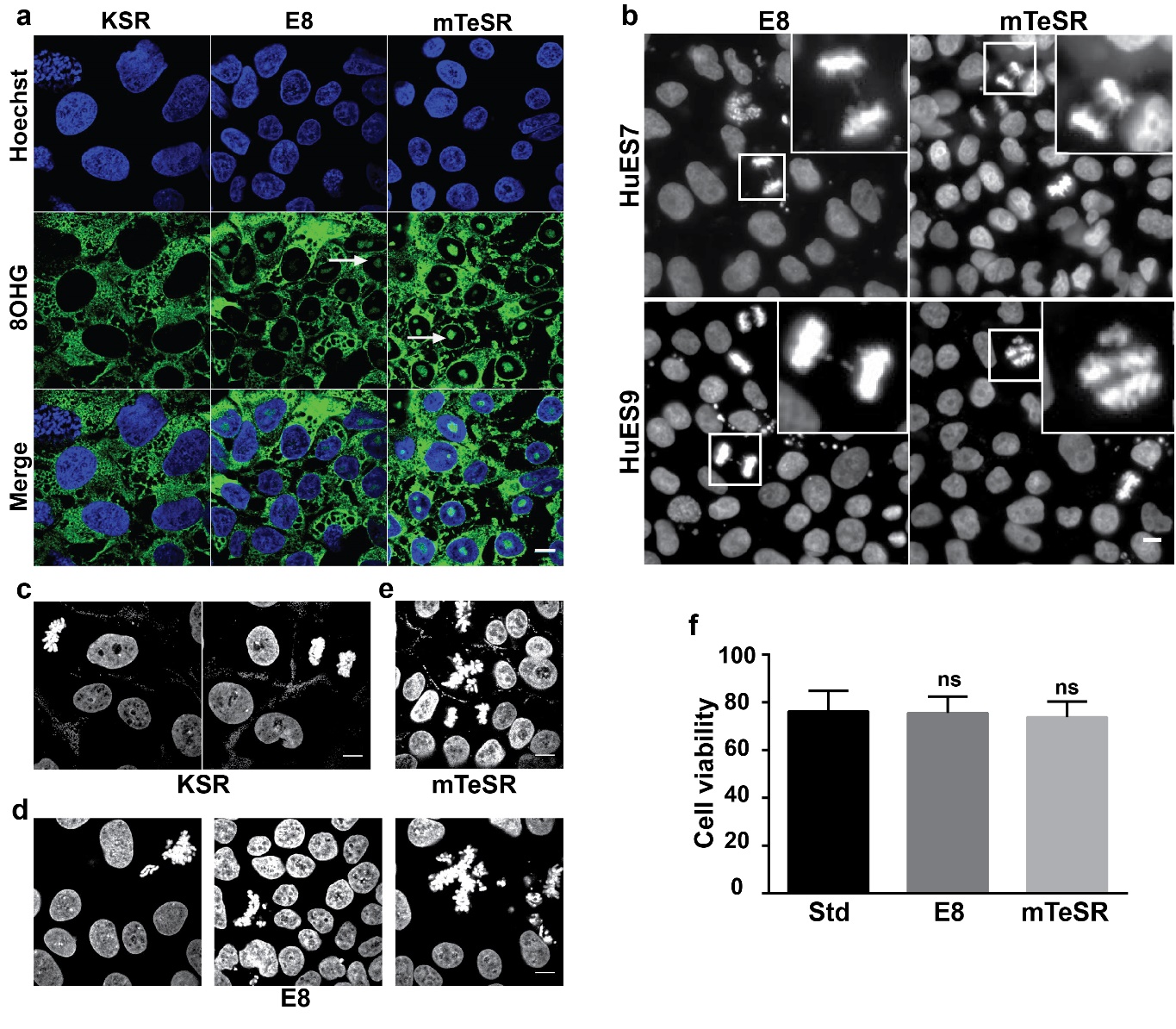
**

(a) Representative images showing higher nucleolar 8-OH guanosine immunostaining of HPSCs in mTeSR media followed by E8 and negligible levels in KSR media. Nuclei are counter-stained with Hoechst 33342. Prominent nucleolar marking with 8-OHG antibody especially in mTeSR and E8 is shown by arrows. (b) Representative wide-field microscopy images of Hoechst-stained HPSCs in E8 and mTeSR showing aberrant mitotic figures. (c) Confocal images of Hoechst staining of HPSCs in KSR media showing normal mitotic figures. (d) Confocal images of Hoechst staining of HPSCs in E8 and (e) mTeSR showing aberrant mitotic figures. (f) Cell viability of HPSCs in the three media; n=3. Pooled data from all the cell lines represented as mean ± SEM. Unpaired t-test with Welch’s correction. All scale bars represent 10μm.

**Supplementary figure S4 related to figure 3: There are more number of dividing cells in
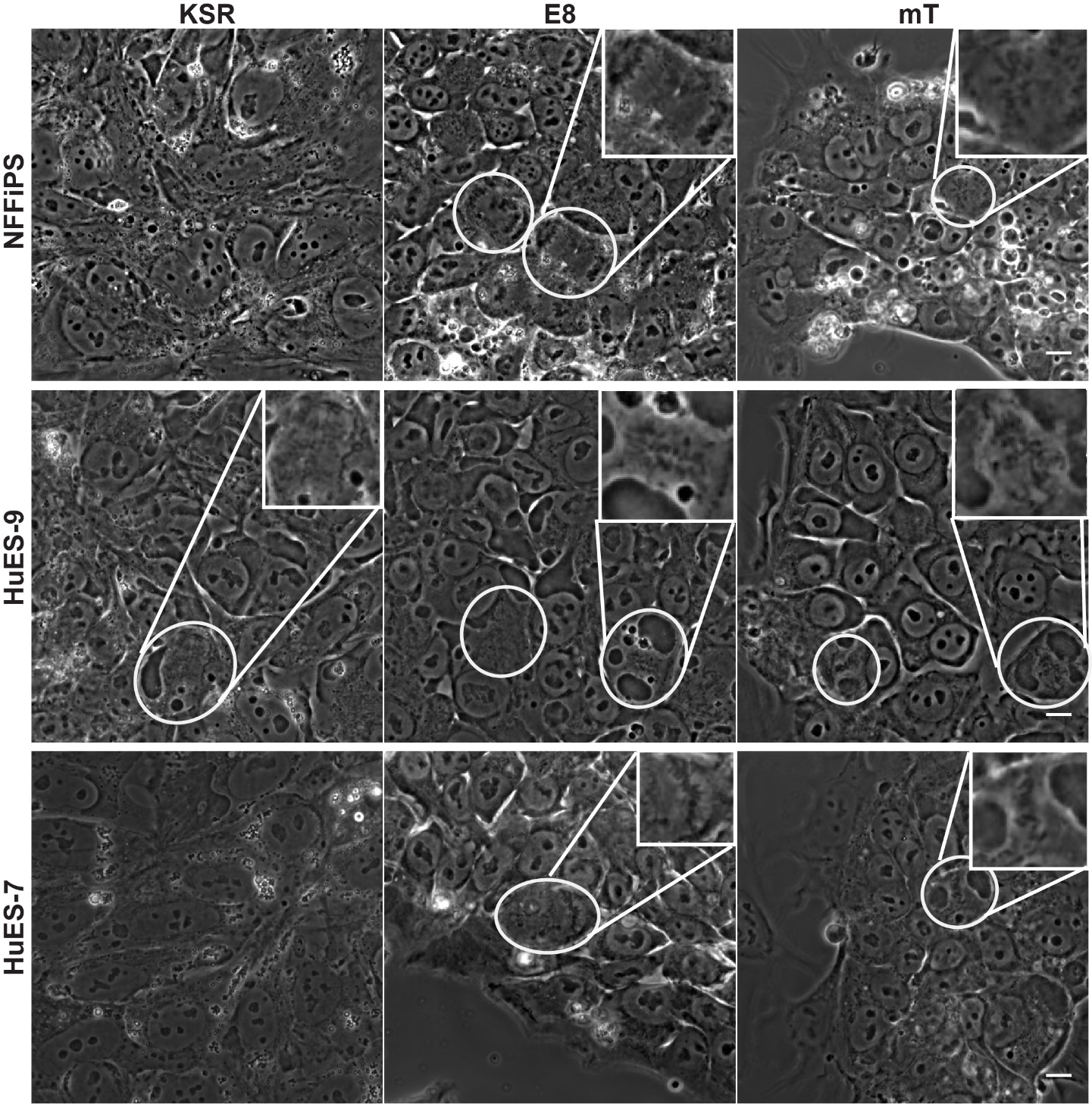
HPSCs in E8 and mTeSR than in KSR media**

Phase contrast images of three different live HPSC cultures showing higher number of dividing cells as marked by the circles, in E8 and mTeSR when compared to KSR. Insets show a magnified version of the corresponding section in the image. All scale bars represent 10μm.

**Supplementary table S1: Effect of high bFGF concentration on increasing ROS levels in KSR and antioxidants on ROS levels in E8 and mTeSR**

| Compound | Reference | Normalized ROS levels | | p-value | | n | |
| --- | --- | --- | --- | --- | --- | --- | --- |
|  |  | **E8** | **mTeSR** | **E8** | **mTeSR** | **E8** | **mTeSR** |
| 250µM GSH | **(Descalzo et al., 2016)** | **1.58±0.16** | **1.75** | ***** | **-** | **3** | **2** |
| 1mM N-acetyl cysteine | **(Ji et al., 2014)** | **1.8** | **1.38±0.25** | **-** | **ns** | **2** | **3** |
| 1µg/ml  Tocopherol acetate | **(Descalzo et al., 2016)** | **1.12±0.12** | **1.02±0.29** | **ns** | **ns** | **3** | **3** |
| 1µg/ml α-Tocopherol | **(Descalzo et al., 2016)** | **1.25±0.2** | **0.89±0.08** | **ns** | **ns** | **3** | **3** |

| **Compound** | **Reference** | **Normalized ROS levels** | **p-value** | **n** |
| --- | --- | --- | --- | --- |
| **100ng/ml bFGF** | (**Shi** et al., 2015) | **1.28±0.08** | ***** | **3** |

- The top panel in blue shows the effect of high concentrations of bFGF for 48 hours on ROS levels of HPSCs cultured in KSR.
- Antioxidants listed in the lower panel were added to examine their effect on ROS levels in E8 and mTeSR media for three passages. Data normalized to their respective untreated controls and represented as mean ± SEM.

**Supplementary table S2: QC of Whole exome sequencing**

|  | **Number of reads** | **Mapped reads, both in pair** | **Mean Mapping Quality**  **(per chromosome)** |
| --- | --- | --- | --- |
| HuES9 KSR | 88,03,716 | 6,917,504 / 78.57% | 31.96 |
| HuES9 E8 | 146,83,535 | 12,015,686 / 81.83% | 50.6 |
| HuES9 mT | 261,06,531 | 21,618,141 / 82.81% | 50.74 |
